# Fatal *Naegleria fowleri* Meningoencephalitis, Italy

**DOI:** 10.3201/eid1010.040273

**Published:** 2004-10

**Authors:** Paola E. Cogo, Massimo Scaglia, Simonetta Gatti, Flavio Rossetti, Rita Alaggio, Anna Maria Laverda, Ling Zhou, Lihua Xiao, Govinda S. Visvesvara

**Affiliations:** * University of Padova, Padova, Italy;; † University-IRCCS S. Matteo, Pavia, Italy;; ‡ Hospital of Monselice, Padova, Italy;; § Centers for Disease Control and Prevention, Atlanta, Georgia, USA

**Keywords:** Meningoencephalitis, Naegleria fowleri, Amoeba, dispatch

## Abstract

We report the first case of primary amebic meningoencephalitis in Italy, in a 9-year-old boy. Clinical course was fulminant, and diagnosis was made by identifying amebas in stained brain sections and by indirect immunofluorescence analysis. *Naegleria fowleri* was characterized as genotype I on the basis of polymerase chain reaction test results.

Primary amebic meningoencephalitis (PAM) is invariably an acute, often fulminant infection caused by *Naegleria fowleri*, a small, free-living ameba that occasionally infects humans and other mammals. Although rare (≈200 cases have been reported worldwide to date), PAM is frequently fatal, is difficult to diagnose, and does not have effective therapeutic options (*1**–**5*). Although more than half of PAM cases have occurred in the United States, infections have been registered in countries in every continent. In the past, cases of PAM were reported from Europe, especially from the Czech Republic, Belgium, and the United Kingdom (*1*). A single case of fatal opportunistic *Acanthamoeba* encephalitis in a patient with AIDS was documented in Italy in 1992 (*6*). We report the first case of PAM from Italy, diagnosed postmortem in an immunocompetent child who, most likely, acquired the infection in July 2003 after swimming in a polluted water hole of the Po River.

## The Case

A 9-year-old boy was admitted to a hospital in Este, a small town in the Veneto region (northern Italy), with a 1-day history of fever and persistent headache on the right side. The child swam and played in a small swimming hole associated with the Po River in northern Italy 10 days before the onset of symptoms. At the time, the region was experiencing an unusually hot summer. On hospital admission, the patient was febrile (temperature 38°C), with a total leukocyte count of 13,780/mm^3^ and C-reactive protein level of 1.2 mg/L. No meningeal signs were present on physical examination, and results of a cranial computed tomographic (CT) scan without contrast were normal. On day 2, a stiff neck developed, and the patient became progressively sleepy. A lumbar puncture showed cloudy cerebrospinal fluid (CSF) with 2.5 mmol/L glucose, 4.54 g/L protein, and a leukocyte count of 6,800/mm^3^ with 90% neutrophils. Gram-stained CSF smears showed no bacteria, and CSF cultures were negative for bacteria and fungi. On day 3, a blood analysis showed a total leukocyte count 19,600/mm^3^ with 91% neutrophils and a C-reactive protein level of 10.6 mg/L. Empiric therapy with ceftriaxone and corticosteroids was started, and the patient was transferred to the intensive care unit of the pediatric department of Padua University Hospital.

**Figure 1:**
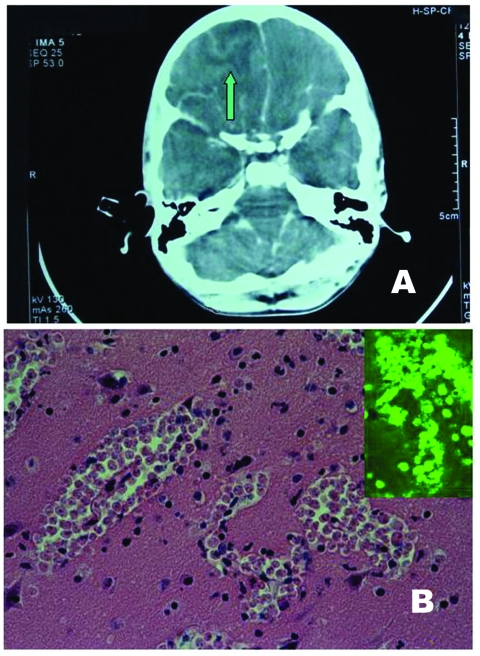
A) Computed tomographic scan: note the right fronto-basal collection (arrow) with a midline shift right to left. B) Brain histology: three large clusters of amebic vegetative forms are seen (H-E stain, x 250). Inset: Positive indirect immunofluorescent analysis on tissue section with anti– *Naegleria fowleri* serum.

Upon admission the child was lethargic, and neurologic evaluation determined a Glasgow Coma Scale score of 9. Treatment with acyclovir and mannitol was started (0.35 g/kg every 6 h). After a few hours, the child became unresponsive to painful stimulation, and he was intubated and mechanically ventilated. Electroencephalogram (EEG) showed decreased electric activity with short, focal, convulsive seizures. Blood and CSF cultures for viruses, bacteria, and fungi were negative, but CSF cell count showed an increase in neutrophils (6,120/mm^3^). The next day, arterial hypertension and tachycardia developed in the patient. A repeat CT scan showed a lesion in the right frontal lobe and diffuse cerebral edema (Figure 1A). Approximately 1 hour later, severe anisocoria (10 mm right and 7 mm left) developed, followed by fixed mydriasis. EEG showed isoelectric activity, and the patient was pronounced dead 6 days after onset of symptoms.

Autopsy was performed 30 hours postmortem after obtaining permission from the parents. Body development was normal, and no chronic diseases were evident. The gastrointestinal tract, liver, and other abdominal viscera showed no abnormalities. Gross neuropathologic findings consisted of swollen and edematous brain with opaque and congested leptomeninges. A cerebellar tonsillar herniation and soft, easily breakable frontal lobes were found. Coronal sections of cerebral hemispheres showed diffuse and multiple foci of hemorrhagic necrosis in both gray and white matter. A preliminary histopathologic examination showed a massive and diffuse inflammatory infiltrate, characterized by a high number of neutrophils, few eosinophils or macrophages, and numerous large clusters of cells that morphologically resembled amebic vegetative forms, tentatively classified as *Entamoeba* or *Naegleria*.

When the case was referred to the parasitology laboratory (Infectious Diseases Department, Pavia University Hospital), a definitive diagnosis of PAM was made on the basis of morphology of amebic trophozoites, which exhibited a conspicuous karyosome, a vacuolated cytoplasm, and a mean diameter of 10 µm to 12 µm. Amebic trophozoites were present in high numbers, often located in perivascular spaces (Figure 1B), and cysts were conspicuously absent, a characteristic feature of PAM.

Formalin-fixed, paraffin-embedded slides and frozen brain specimens were also sent to the Centers for Disease Control and Prevention (Atlanta, GA) for final identification and characterization of the species. Based on the reactivity of amebas in tissue sections with anti–*Naegleria fowleri* serum in indirect immunofluorescent (IIF) analysis (Figure 1B, inset), the etiologic agent was identified as *N. fowleri*.

**Figure 2:**
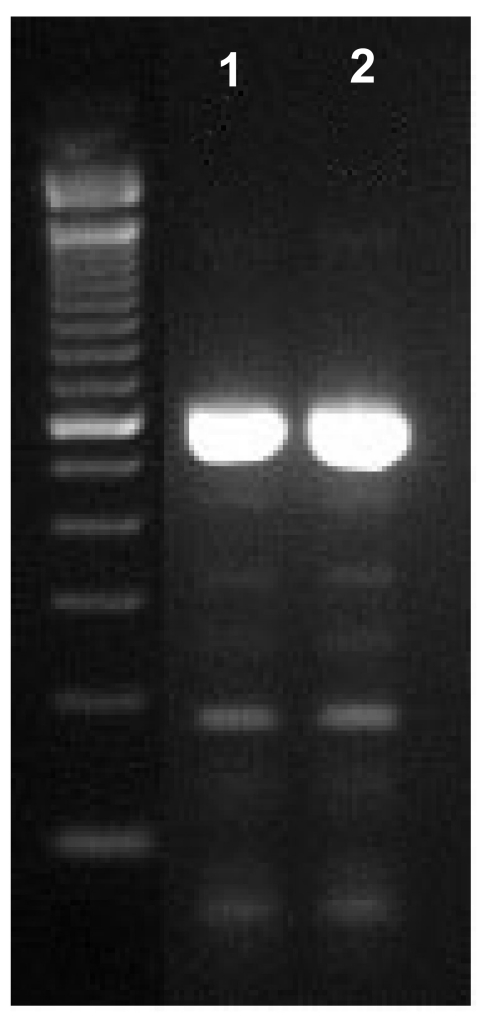
Identification of *Naegleria fowleri* in the brain specimen from the Italian child by polymerase chain reaction analysis of the internal transcribed spacer of the rRNA gene. Lane 1: DNA from infected brain of the patient; lane 2: DNA from CDC:V236 culture, a positive control for *N. fowleri*.

DNA was extracted from frozen brain tissue. The infected brain tissue was initially subjected to alkaline digestion with 66.6 µL of 1 mol KOH and 18.6 µL of 1 mol dithiothreitol at 65°C for 15 min, neutralized with 8.6 µL of 25% HCl (vol/vol), then buffered with 160 µL of 2 mol Tris-HCl (pH 8.3). DNA was extracted with 500 µL of phenol:chloroform:isoamyl alcohol (25:24:1, vol/vol/vol) (Invitrogen Inc., Carlsbad, CA) and purified with QIAamp DNA stool mini kit (Qiagen Inc., Valencia, CA). A fragment (≈600 bp) of the internal transcribed spacer of the rRNA gene was amplified by using primers NF-ITS-F1 [5´-GAC TTC ATT CGT TCT TGT AGA-3´] and NF-ITS-R1 [5´-CTC TTG CGA GGT CCA GAC-3´] (*7*). Genotype of *N. fowleri* was determined on the basis of DNA sequencing and sequence comparison with published data (Figure 2). DNA sequencing of the &8776;600-bp polymerase chain reaction (PCR) product further showed that the ameba was identical to the previously described genotype I or the widespread variant, which was previously found in France, Hong Kong, and the United States (*7*).

## Conclusions

As the clinical and epidemiologic history demonstrate, our patient contracted PAM caused by *N. fowleri* 10 days after swimming and diving in polluted river water in Italy during the unusually hot summer. He displayed characteristic, though not strictly specific, clinical features of PAM (*1**,**2*): 1) hyperacute clinical course; 2) unrelenting signs and symptoms of meningitis and encephalitis, the latter confirmed by CT imaging; 3) high levels of peripheral leukocyte count, mainly polymorphonuclear leukocytes; 4) cloudy CSF with leukocytes, hyperproteinosis, low glucose level, and absence of bacteria and fungi; 5) rapid worsening of disease, leading to death within a week. Gross pathologic and histologic findings confirmed the clinical suspicion.

None of the patient’s friends and relatives who swam in the same water hole on the same day became ill. Nasal swabs from all of them were negative for amebas, which confirms that fatal *N. fowleri* infection is rare.

Previous epidemiologic studies, conducted in Italy on warm water and thermal mud, failed to isolate *N. fowleri*, although they isolated strains of two other *Naegleria* species, *N. italica* and *N. australiensis*, which are experimentally pathogenic to mice (*8**–**11*). Therefore, we initially hypothesized that this case could be caused by one of these species; however, *N. fowleri* was identified by IIF analysis, and PCR confirmed it as genotype I.

This case is the first diagnosed occurrence of PAM in Italy. Few clinicians and microbiologists in Italy are aware of the disease and the potential danger presented by other free-living, pathogenic species of amebas, such as *Acanthamoeba* and *Balamuthia*. Consequently, other cases may have gone undiagnosed.

We emphasize that environmental conditions, in particular, the unusually hot summer of 2003 in Italy and other European countries, have strongly contributed to increasing the surface temperature of natural, open-air basins, such as rivers, lakes, and ponds. According to the forecast by a United Nations scientific advisory panel, global temperature will rise 0.8°C–3.5°C by the year 2100 if production of greenhouse gases is not reduced. An increase in surface temperature will create ideal niches for the thermophilic *N. fowleri* (*1**,**2*). Persons who bathe, swim, or dive in pools or freshwater natural basins will increase their chances of coming into contact with *N. fowleri* and contracting PAM.
